# Evaluating Image Quality Metrics as Loss Functions for Image Dehazing †

**DOI:** 10.3390/s25154755

**Published:** 2025-08-01

**Authors:** Rareș Dobre-Baron, Adrian Savu-Jivanov, Cosmin Ancuți

**Affiliations:** Faculty of Electronics, Telecommunications and Information Technologies, Polytechnic University Timisoara, 300006 Timisoara, Romania; rares.dobre-baron@student.upt.ro (R.D.-B.); adrian.savu-jivanov@upt.ro (A.S.-J.)

**Keywords:** image quality assessment, image metrics, loss functions, dehazing

## Abstract

The difficulty and manual nature of procuring human evaluators for ranking the quality of images affected by various types of degradations, and of those cleaned up by developed algorithms, has lead to the widespread adoption of automated metrics, like the Peak Signal-to-Noise Ratio (PSNR) and the Structural Similarity Index Metric (SSIM). However, disparities between rankings given by these metrics and those given by human evaluators have encouraged the development of improved image quality assessment (IQA) metrics that are a better fit for this purpose. These methods have been previously used solely for quality assessments and not as objectives in the training of neural networks for high-level vision tasks, despite the potential improvements that may come about by directly optimizing for desired metrics. This paper examines the adequacy of ten recent IQA metrics, compared with standard loss functions, within two trained dehazing neural networks, with observed broad improvement in their performance.

## 1. Introduction

### 1.1. Image Dehazing

Image dehazing is a common high-level vision task with multiple unique applications and challenges. Fog, haze, smoke, and similar phenomena are natural sources of image degradation in multiple domains, from satellite images to automotive camera perception. Sensors, especially cameras and imaging systems, often struggle to capture clear images in environments with atmospheric disturbances like fog, haze, or smoke. Dehazing techniques are essential for improving the quality of images captured by these sensors, enhancing the accuracy of downstream applications such as object detection, autonomous navigation, surveillance, and environmental monitoring.

While dense haze can potentially obstruct entire segments of the input image, possibly eliminating vision entirely in severe instances, even medium and low-level haze have a negative impact on the ability of both humans and computer vision (CV) systems to accurately detect and classify objects (see [1,2,3]). Besides the erasing of segments (necessitating probabilistic reconstructions only possible with deep learning methods), haze distorts the contrast and luminosity of images, decreasing the former and increasing the latter, thereby pushing all pixel colors towards white and flattening the color histogram.

Due to the complexity, heterogeneity, physical, and natural aspect of this distortion, haze removal is considered a high-level and difficult image reconstruction task, and the overwhelming success of deep learning (DL) methods, which are present in essentially all CV applications, has been even more pronounced for dehazing tasks.

The first viable methods for removing haze were based on neural networks (NNs) [4,5,6,7] and relied on various approximations of the environment, such as the dark channel prior (DCP) [4]. Recent methods increasingly leverage and solely rely on the statistical and computational power of approaches such as transformers [8], diffusion models [9], MAMBA methods [10], and others.

A common issue with many dehazing models is their choice of loss functions. Despite the unique challenges of the high-level vision task of dehazing, most neural networks in this area are still trained using standard loss functions from the current machine learning paradigm—such as L2 (mean squared error), L1 (mean absolute error), and their variants—as well as architecture-specific losses like adversarial losses. These approaches come with well-known statistical and empirical limitations. More recently, some models have adopted loss functions based on widely used image quality assessment (IQA) metrics—namely the Peak Signal-to-Noise Ratio (PSNR) and the Structural Similarity Index Measure (SSIM), along with their variants. While these are an improvement, they too have inherent drawbacks.

### 1.2. Image Quality Assessment Metrics

Image quality assessment (we will be focusing on the full-reference variety) is a domain of image processing that seeks to develop methods and metrics that can better approximate the judgments given by real human evaluators that are related to the quality of images affected by distortions [11,12].

There are multiple motivations behind this domain. The first is the practical issue of requesting human evaluation for image quality being expensive, time-consuming, wholly impractical for quickly iterating work, and occasionally fraught. The second motivation is the need to capture the idiosyncrasies of human perception and its mechanisms (background vs. foreground, sensitivity to certain colors over others, etc.) [13], which have very specific evolutionary adaptations and translations into subjective opinion.

Due to their origin in mathematical distance measures [14], classical measures for quantifying distortions of known images, while straight-forward and expressive, were not powerful enough to capture the mentioned idiosyncrasies, and furthermore they simply failed to accurately correspond to human scores or rankings. It is a known result that the MSE measure excessively penalizes small rotations and translations that humans are generally not concerned with, and they give similar scores to distortions noticeably different in aspect and intensity [15].

The PSNR is a standard objective measure that is inherited from the signal processing domain, which again fails to correspond to human perception. The SSIM, a subjective measure, was created specifically to address issues with the PSNR and MSE, taking into account features of the entire image (as opposed to only local features), with its multi-scale extension MS-SSIM being especially popular for this purpose [16]. However, in spite of the enduring popularity of the PSNR and SSIM, the measures still proved inadequate for many applications, and examples of discrepancies between their rankings and human perception are common. As such, a rich literature of additional IQA metrics has been formed, each trying to take into account certain aspects of human vision, formalizing them into an approximate mathematical form or using the vision of deep learning solutions. See Section 3 for detailed discussions of several of these metrics.

### 1.3. IQA Metrics as Objectives

A vast majority of the metrics in question have only been used for, as their name implies, assessments. They are treated as static standards by which the outputs of algorithms are judged (usually, though not always, utilizing a reference). While no doubt useful, there is a missed opportunity for using these measures as objectives in themselves for the algorithms in question. As the usual objective of operations such as denoising, upscaling, and low-light enhancement (LLA) directly serve the needs of humans, optimizing for a closer proxy of their judgments is a reasonable course of action, as previously explored by Ding et al. [17].

There is a valid concern related to using such proxies for human perception as direct objectives for the networks. Goodhart’s Law (in this context the Regressional and Extremal variants [18]) tell us that placing optimization pressure on the proxy of a real metric will likely, at some point, especially at the extremes of either metric, lead to a divergence between the two. The network would learn ways of maximizing the proxy in a way that reduces the output’s fitness to the real metric (human perception). The examples with the PSNR and SSIM mentioned in the previous example are such instances, and the additional complexities of the metrics to be showcased might exacerbate such an issue. Our results, however, have thankfully not borne out this prediction.

Furthermore, a large degree of image enhancement work is performed so that the cleaned images are fed into different algorithmic systems, which perform separate tasks. For example, dehazing solutions are often used within broader object detection and classification networks, as is the case for automotive applications, to various degrees of embedment. While it is possible that human metrics as objectives might be unnecessary or potentially harmful for the purposes of separate networks and their tasks, our results show that networks trained on human metrics show improvements in general readability, as well as values of the PSNR and SSIM. This is an area for further research.

### 1.4. Contributions

Extending the work of Ding et al. [17] on low-level image tasks (denoising and deblurring) to a high-level task (dehazing), we have innovated in the following manner:

- Training two dehazing architectures (one older and one near State-of-the-Art) using 17 different loss functions, 7 standard and 10 novel, based on recent image quality assessment metrics.
- Proving the efficacy of IQA metric-derived objectives for dehazing tasks relative to classic loss functions and demonstrating the viability of this approach for future high-level image processing tasks.

The paper is organized into the following sections: Section 1 gives an overview of the research strands related to the paper at hand and our contributions. Section 2, titled “Related Work,” gives pointers of the methods under discussion, as well as previous iterations of our main idea. In Section 3, “Methods,” we describe the networks and the metrics under consideration. Section 4 presents the results obtained on real-world dehazing datasets. At the end we give a discussion of further research and conclusions in Section 5.

## 2. Related Work

There exists a large body of literature on dehazing methods, employing essentially every major innovation in the field of (convolutional) neural networks. While initial models were basic CNNs or U-Nets, the first major development was the use of a dark channel prior (DCP) [4], which utilized a mathematical approximation of the manner haze that distorts visual perception, namely the atmospheric scattering model:

(1) I(x)=J(x)t(x)+A(1−t(x))

where I(x) is the observed hazy image, J(x) is the scene radiance (ideal, clean image), *A* represents the global atmospheric light, and t(x) is the transmission matrix, which decreases exponentially with distance. Most early, basic CNN methods utilized this model (e.g., [19,20,21,22]).

In line with developments in other fields [23], the ensuing methods employed less priors about the physical means of dehazing the distorted image, and instead they opted to use powerful, general-purpose constructions, including GANs [24,25], the attention heads of transformers [8], the diffusion-based generative abilities of diffusion models [9], the use of state space nodels (SSMs) [10], and many others [26].

The IQA literature is similarly well-developed. Numerous variants of the PSNR and SSIM such as the multi-scale SSIM (MS-SSIM) [16] exist, as well as metrics employing various human priors, like PSNR-HVS [27] (based on the JPEG standard, itself derived from human perceptions of frequency [28]) or NLPD [29] (based on the human vs. digital range of color frequencies). The use of neural networks for object detection and classification, in some cases exceeding that of real humans, has also given inspiration to metric designers, which have utilized pre-trained segments of well-known networks like Alex-Net [30] and VGG [31] to derive similarities between images fed to them based on the activations of those segments. The use of such losses was already common in broader networks which contained detection/classification/segmentation auxiliary tasks.

While the use of the PSNR and SSIM as losses are widespread, the use of more advanced metrics for this same purpose has only been previously explored by Ding et al. [17], although only on low-level tasks: denoising, deblurring, super-resolution, and compression. Some metrics gave strange results that lack utility; however, a couple of metrics (including the NN-derived LPIPS [32] and DISTS [33], as outlined below) proved themselves capable of serving as objectives for such tasks. It seems, however, that the findings of the paper have not been replicated or meaningfully integrated into the broader ML computer vision paradigm. Our work is the first to extend those results to higher-level tasks, namely dehazing, and proves their feasibility in such applications, as the unreliable results did not manifest when the network was sufficiently complex (see Section 4).

## 3. Methods

### 3.1. Networks

In order to assess the adequacy of the metrics in question as losses for multiple kinds of networks, two dehazing solutions were chosen for this purpose.

#### 3.1.1. AOD-Net

AOD-Net [34] is an important milestone in the use of DCP, as it successfully simplified the atmospheric scattering equation into

(2) J(x)=K(x)I(x)−K(x)+b

(3) $$K\left(x\right)=\frac{\frac{1}{t(x)}\left(I\left(x\right)-A\right)+A-b}{I(x)-1}$$

K(x) is determined from a simple convolution neural network with skip connections and concatenation layers, and *b* is a constant bias. This gave the network a compact size and a fast training speed. Models before AOD-Net had tried to estimate both t(x) and *A* at the same time. AOD combines the two into the more convenient K(x) term.

AOD-Net is a small, fast-training network which can handle any size of input image, and it utilizes physical priors in the form of the DCP.

#### 3.1.2. UVM-Net

UVM-Net [10] was chosen as it is closer to current NN design sensibilities. It utilizes selective state space models implemented in the popular MAMBA module, with performance similar to and in some cases exceeding that of transformers in speed and memory, for a wide variety of tasks [35].

It is a large, near-SOTA model with a U-Net structure, which can also be trained for a variety of other image enhancement tasks, as outlined in the original paper.

### 3.2. Metrics

We can broadly classify the loss functions we will use into two categories: the classic loss functions, which are already present and widely used in the literature for this purpose (including the PSNR and SSIM), and the novel, metric-derived loss functions, which we introduce in this analysis.

#### 3.2.1. Classic Loss Functions

The classic loss functions under analysis are the L2 Loss (MSE), the L1 Loss (MAE), the Smooth L1 Loss, the Huber Loss, PSNR, SSIM, and MS-SSIM. As the last three are ideally maximized, the losses are given as the negative of those metrics.

##### Mean Squared Error (MSE)/Quadratic Loss/L2 Loss

The L2 loss is the standard loss function of most ML-based algorithms. It intuitively resembles Euclidean distance and strikes a reasonable balance between sufficient freedom (to prevent overfitting for errors close to 0) and sufficient strictness. However, in image processing applications, it tends to produce an averaging, smoothing effect over the whole image, a consequence of the mathematical fact that, when taking the sum of all errors into account, the average value between all of them gives the smallest L2 value. The L2 loss is also very sensitive to outliers, which is a problem that can lead to overfitting. It is for this reason that L2 has often been abandoned in favor of L1 and its less restrictive variants.

##### Mean Absolute Error (MAE)/L1 Loss

The L1 loss has increased in popularity as its strictness and mathematical formulation discourages the averaging effect of L2. However, it can occasionally prove too strict near 0, with a sharp shape, giving unstable, overfitted results, and as such it is either used sparingly or as a small component of a larger loss function. Often its smoother, less harsh variants, such as Smooth L1 and Huber, are used instead.

##### Smooth L1 Loss

Smooth L1 is superficially similar to L2 when the error is small (prohibiting the smoothing effect), but it is similar to L1 for medium errors, giving better correction abilities when the resulted values are moderately far away from the desired ones, without being outliers. It is also less sensitive to large outliers than L2. This combination of advantages of L1 (robustness for large errors) and L2 (less harsh errors and good convergence for values close to reality) has made Smooth L1 very popular [36].

##### Huber Loss

The Huber Loss is very similar to Smooth L1, carrying all of its advantages, while giving greater freedom to the slope of the L1 portion. In our case the formula is equivalent to that of Smooth L1.

The four previous loss functions are all fidelity-based [26], supervised, and regression-based [37], and they are often used in the literature for a wide variety of tasks.

##### PSNR

The standard Peak-Signal-to-Noise-Ratio metric is the most widely used metric for image processing result evaluation, being an objective measure of an image’s quality. Its calculation includes the MSE of the image and is thus subject to similar distortions as L2. Even though it has lost ground to the SSIM, as the PSNR does not correspond to human visual perception very well, encouraging averages and smoothing, it is still an important benchmark. Its use as a loss function, however, has been limited. The loss function used is the PSNR.

##### SSIM

The Structural Similarity Image Metric [38] was created out of a need for better image processing result evaluation than the PSNR, specifically one that would more accurately reflect human visual perception. The SSIM, being a subjective measure, has been widely successful in this regard, having better correlation than the PSNR with subjective image quality scores across a variety of degradations. It is structurally similar to MSE [39]. The structural loss function used is 1−SSIM.

##### MS-SSIM

Multi-scale SSIM (MS-SSIM) [16] is SSIM calculated on multiple scales and sampling frequencies, providing better results than the SSIM, and it has occasionally been used as a loss function. The structural loss function used is 1−MS−SSIM.

#### 3.2.2. IQA Loss Functions

Out of the many IQA metrics in the literature, we have chosen the following for our analysis: HaarPSI [40], PSNR-HVS [27], CW-SSIM [41], LPIPS [33], DISTS [32], MSSWD [42], NLPD [29], PIEAPP [43], WADIQAM-FR [44], and TOPIQ-FR [45]. They are all full-reference metrics, making them appropriate for comparisons to a ground-truth image.

##### HaarPSI

HaarPSI [40] is a metric based on the Haar wavelet, taking into account visual distortions observable by a human in the frequency domain. It utilizes two Haar wavelet transforms over the image, as well as a similarity function similar to the SSIM. The two Haar wavelets are a low-pass scaling filter and a high-pass scaling filter, respectively. The Haar wavelet is used to extract local information from the image. The formula of the metric contains similarities to the SSIM in structure, using the Haar-filtered values of the image instead of the variance, and also uses a function similar to a sigmoid activation function to introduce non-linearities into the metric. It was chosen for our analysis because it has been proven to be consistent with human visual perception. Its use as a loss function is novel. The loss function used is −HaarPSI.

##### PSNR-HVS

PSNR-HVS [27] is a metric based on the human visual system (HVS) and utilizes the discrete cosine transform, having greatly improved the correlation with human perception across a variety of distortions. PSNR-HVS is a PSNR that is adjusted to take into account the different frequencies of an image and their importance to human perception, based on the quantization table used in the JPEG standard [28]. The metric was chosen for our analysis because of its deep connection with standard results of human visual perception as applied to the computer vision domain. Its use as a loss function is novel. The loss function used is −PSNR−HVS.

PSNR-HVS-derived metrics (PSNR-HVS-M [46], which expands PSNR-HVS by including a contrast sensitivity function (CSF) that also takes into account the human perception of visual wavelengths that are close together, and PSNR-HA and PSNR-HMA [47], complex metrics utilizing corrected images approximating the original image and PSNR-HVS) have also been attempted as loss functions. All have led to quickly exploding gradients, likely due to complex and poorly differentiable if-laden calculations for PSNR-HVS-M including a four-pronged if condition and likely due to multiple divisions by small numbers present in their formulas for PSNR-HA and PSNR-HMA. As they all require division by a difference between two almost identical matrices, they are excluded from our analysis.

##### CW-SSIM

The complex wavelet structural similarity (CW-SSIM) index [41] was created to address issues of the SSIM’s sensitivity to small transformations, such as rotations and translations. CW-SSIM posits that these distortions induce consistent phase changes in the local wavelet coefficients of the image and can thus be ignored as they do not change the underlying structure of the image. The metric was chosen for our analysis due to its mathematical similarities to the SSIM and greater emphasis on the underlying structure of the image. It has previously been used as a loss function by Ding et al. [17] for deep, encompassing distortions. The loss function used is −CW−SSIM.

##### LPIPS

The learned perceptual image patch similarity (LPIPS) metric [33] was the first to recognize the potential of vision models for use in image quality assessments. After the advent of classical CNN-based DL architectures exceeding the average capabilities of humans for certain recognition tasks, it has been observed that the interior activations of these networks between similar inputs are themselves similar, specifically in ways that are salient and reminiscent of human visual perception. The activations of trained VGG networks, for instance, have often been used as a perceptual loss to augment pre-existing losses. The potential of this perceptual loss to assess image quality as well was recognized by Zhang et al., leading to the creation of this metric based on the activations of a static Alex-Net model [30] (or others), which uses L2 distances between them. While somewhat slow, as the calculation of this metric includes an inference of a whole (though relatively compact) neural network, this bottom-up metric and its derivatives can be thought of as approximations of an idealized human perception metric, one which can directly look inside the relevant neural activations of a person to gauge the similarity they perceive between two images. These metrics do use an artificial, as opposed to a natural, neural net, which nevertheless has greater capacity of detection and recognition than its biological inspiration. LPIPS begat several more such DL-derived measures. This metric was chosen due to the previously outlined reasons. It has previously been used as a loss function by Ding et al. [17], with good results. The loss function used is −LPIPS.

##### DISTS

The deep image structure and texture similarity (DISTS) metric [32] is one of a series of DL-derived metrics, like LPIPS above. It is based on the VGG architecture [31] and innovates on previous such measures by taking into account textural variation. This metric is not concerned with changes that preserve the local texture of an image (such as grass of the same color, length, and density), which is often distorted in a manner that is not noticeable, especially in corrupted image recovery (where the metric performs better than LPIPS). It is structurally and mathematically similar to SSIM. The metric was chosen as minor changes which keep underlying texture intact are not relevant for the dehazing process, a characteristic that might potentially boost performance. It has previously been used as a loss function by Ding et al. [17], with good results. The loss function used is −DISTS.

##### MSSWD

The multi-scale sliced Wasserstein distance (MSSWD) [42] was conceived with considerations of the global color profile. Instead of comparing local patches of color, as in a majority of other metrics, the MSSWD instead considers the similarity of the overall coloration between images to be more important than strict local correspondence of position. It is a complex algorithm and not strictly a distance, though it does act mathematically as a metric: it applies spatial filters to both images, flattens and sorts each channel of the image, and compares the resulting vectors using the L1 distance. This ensures that shifts in the global features of the images (color especially) are prioritized over local features. The metric was chosen because a loss of contrast and increased lumination (distorted color profile) is a large problem inherent to dehazing, and because color discrepancies appeared when training our chosen models using the classic loss functions. Its use as a loss function is novel. The loss function used is MSSWD.

##### NLPD

The normalized Laplacian pyramid distance (NLPD) [29] was an incidental metric developed for the purpose of rendering photographic images in a manner that better conforms to the color field of human vision. The range of colors that an average human sees is different and much wider than that projected by a screen, necessitating a more complex rendering which takes into account the successive, Laplacian-like images that are processed by HVS. This problem was formalized as a constrained optimization problem involving the minimization of the NLPD, which involves successive downscaling and filtering of the images, followed by several weighted arithmetic averaging steps. The metric was chosen as its focus on color and real human perception could potentially be useful in haze removal. It has previously been used as a loss function by Ding et al. [17], with good results. The loss function used is −NLPD.

##### PIEAPP

The perceptual image-error assessment through pairwise preference (PIEAPP) metric [43] sought to solve a common problem in IQA metrics: the fact that those derived from human rankings of images were unreliable due to arbitrary and unclear scoring by human evaluators. In contrast, PIEAPP constitutes a simple neural network that was trained on pairs of images to predict the probability that one of the other would be preferred by human evaluators, as pairwise preferences were found to be much more reliable and easier for the evaluators. The metric was chosen due to its sensitivity, and its training data were derived from empirical and robust human evaluations on a variety of common distortions. It has previously been used as a loss function by Ding et al. [17], with extremely poor results. The loss function used is −PIEAPP.

##### WADIQAM-FR

The weighted average deep image quality measure for FR IQA (WADIQAM-FR) [44] is a DL solution that trained a complex neural network utilizing feature extraction and weighted pooling on a pair of distorted and reference images to determine image quality. It is otherwise a straightforward metric. The metric was chosen due to its simplicity and representation of a larger class of IQA metrics. Its use as a loss function is novel. The loss function used is −WADIQAM−FR.

##### TOPIQ-FR

TOPIQ [45] is an improvement over previous DL-based metrics. Instead of taking the parallel approach of successive resizing used by metrics such as the SSIM, or the bottom-up DL approach of DISTS or LPIPS, TOPIQ combines these conceptions into a top-down approach: image features are extracted and combined to form multiple scales of the image in the trained network. The metric was chosen due to its improvement and uniqueness over others in its class, showcasing good and robust previous results, simplicity, and representation of a larger class of IQA metrics. Its use as a loss function is novel. The loss function used is −TOPIQ−FR.

## 4. Results

### 4.1. Metric Details

A majority of the classical loss functions are standard PyTorch 2.7.0 loss functions. PSNR, SSIM, MS-SSIM, HaarPSI, and LPIPS came from the piqa image metric library [48]. PSNR-HVS came from the psnr_hvsm library [49]. All other metrics originated from the IQA-PyTorch (pyiqa) library [50], and they were used as losses. All settings were standard.

### 4.2. Architecture Details

Both networks were trained without modification using the datasets recommended by each author. Due to the requirements of several metrics, an additional normalization step was added at the end of UVM-Net.

AOD-Net gave outputs of the same size as the input. UVM-Net, however, compressed each image into 512 by 512 pixel squares. As such, the output images were resized to their respective input sizes using PIL bicubic interpolation, and they were saved as .jpg files. This degradation affected all images uniformly for the UVM-Net outputs.

### 4.3. Training Details

AOD-Net was trained using an NVIDIA GTX 3070 GPU, while UVM-NET was trained using two NVIDIA GeForce RTX 3090 Ti GPUs. The networks were trained for 40 epochs each. AOD-Net used Adam optimization, and UVM-Net used SGD. The batch size was eight for AOD-Net and one (stochastic) for UVM-Net. The learning rate was $10^{-4}$ for both models.

### 4.4. Results

Both networks were tested on standard real-world dehazing datasets I-Haze [51], O-Haze [52], and NH-Haze [53,54] for interior, outdoor, and non-homogeneous haze, respectively.

### 4.5. Results Discussion

Two trends are apparent from Table 1: firstly, the IQA-derived metrics usually perform better than the classic loss functions for every dataset, especially for the more complex UVM-Net architecture. With exceptions that showcase the nevertheless enduring viability of the simpler and well-established loss functions (such as PSNR, SSIM, and Huber), the IQA metrics give less distortions and significantly better results in some cases (PSNR-HVS and MSSWD especially). Besides the SSIM performance of the PSNR loss function for AOD-Net, which was evaluated on the I-Haze dataset, the best loss function for any situation was from the group with the IQA metrics, showing their better applicability for this task than the standard loss functions.

PIEAPP stands out for producing both significantly higher scores (e.g., UVM-Net on O-Haze) and significantly lower ones (e.g., AOD-Net on SSIM), though with consistently reduced variance. Notably, it is the only IQA-derived metric that reports color distortions for UVM-Net (see Table 2), which may suggest a mild case of Goodhart’s Law in relation to human perception versus PSNR/SSIM—though the exact impact remains unclear. It is reassuring that many metrics previously flagged as unreliable by Ding et al. [17], such as CW-SSIM, did not produce such anomalies in higher-level tasks.

Secondly, the results show significant heterogeneity. There is little consistency between the loss functions across different datasets and evaluation metrics, highlighting the need for careful testing and validation when selecting loss functions for specific computer vision tasks. Understanding and systematizing the characteristics of these metrics—and the behaviors they promote during network training—is an important direction for future research. That said, aside from the previously discussed PIEAPP, the new metrics performed reasonably well, showing comparable performance and variance to traditional loss functions, with none performing poorly.

The empirical nature of PIEAPP—based on human judgments and especially PSNR-HVS, which reflects real human perception of visual frequencies—gives both metrics an advantage over more abstract, mathematically defined loss functions. Exploring loss functions grounded in empirical human perception is a promising direction for future research, with potential benefits for a wide range of image processing and restoration tasks beyond dehazing.

General strange results and distortions of the AOD-Net network indicate that these metrics are likely not suitable for small, simple networks, as they fail to capture their intricacies. Large networks seem to eliminate this problem, given their increased expressivity.

## 5. Conclusions and Future Work

Our study has established that novel image quality assessment metrics are entirely appropriate for use as training objectives for dehazing neural networks, giving better results than identical networks trained using standard ML and image processing metrics. Previous results [17] had only shown the viability of such metrics for use in low-level tasks, such as denoising and deblurring, with strange Goodhart-derived artifacts and distortions resulting from some of the metrics. The good results, which were almost complete absence of such distortions when using these metrics in a neural network that is complex and lacking in domain-specific abstractions (unlike AOD), for a high-level task such as dehazing all show that applying IQA metrics to the training process of neural networks of this kind is easy and adequate. The modifications explored in this paper can easily be adapted to other high-level tasks, such as rain removal, LLA, and many others, with potentially equally improved results.

Testing each new IQA metric in this manner is something that should be conducted on an individual basis, with checks and adaptations for the tasks at hand. The lack of significantly extended training time (an issue that is proportionally reduced with increased size and complexity of the network being trained) also points towards the potential ease of adaption and extension of this paper’s results.

The most relevant area for future work is the application of these metrics to other complex image processing tasks, such as deraining, recolouring, LLA, and many others. Ding et al. [17] establish the usefulness of some of these metrics for simple tasks and simple networks, while our study has established this for complex tasks and complex networks, where the metrics seem to behave a lot more efficiently and consistently (though generally in similar ranges of performance) than for the former cases.

Future work will also entail finding and designing better image quality assessment metrics that can be tailor-made to each particular task by utilizing known human perceptions of the phenomena that lead to distortion (how the human eye perceives objects obscured by fog, how it filters out rain and dust, etc.). Our findings are preliminary and serve to simply confirm the viability of exploring this domain for future NN-based image processing and computer vision tasks.

## Figures and Tables

**Table 1:** Results on the I-Haze, O-Haze, and NH-Haze datasets on the AOD-Net and UVM-NET models. **Red**, **blue**, and **green** results represent first, second, and third places per architecture, metric, and dataset, respectively.

Methods	AOD-Net						UVM-NET					
	**I-Haze**		**O-Haze**		**NH-Haze**		**I-Haze**		**O-Haze**		**NH-Haze**	
	**PSNR**	**SSIM**	**PSNR**	**SSIM**	**PSNR**	**SSIM**	**PSNR**	**SSIM**	**PSNR**	**SSIM**	**PSNR**	**SSIM**
L2	9.55	0.244	9.95	0.153	8.53	0.041	16.04	0.401	14.16	0.158	11.44	0.105
L1	9.67	0.123	10.16	0.156	8.82	0.051	16.09	0.404	14.15	0.161	11.42	0.106
Smooth L1	9.28	0.245	9.65	0.162	8.29	0.038	16.01	0.399	14.10	0.157	11.42	0.104
Huber	10.09	0.229	10.27	0.178	8.89	0.076	15.94	0.395	14.12	0.154	11.46	0.102
PSNR	9.60	0.258	9.79	0.169	8.46	0.043	16.16	0.410	13.95	0.160	11.28	0.103
SSIM	9.83	0.230	9.54	0.185	8.02	0.082	16.19	0.413	14.03	0.162	11.29	0.105
MS-SSIM	9.27	0.257	9.54	0.168	8.02	0.044	16.20	0.413	14.01	0.161	11.28	0.106
HaarPSI [40]	8.79	0.250	9.09	0.160	7.33	0.048	16.22	0.414	14.07	0.164	11.31	0.106
PSNR-HVS [27]	10.22	0.240	10.53	0.215	9.00	0.103	16.09	0.407	13.89	0.158	11.28	0.101
CW-SSIM [41]	9.02	0.258	9.20	0.166	8.00	0.047	16.22	0.414	14.06	0.163	11.30	0.106
LPIPS [33]	9.42	0.241	10.40	0.181	8.95	0.054	16.23	0.413	14.09	0.164	11.33	0.107
DISTS [32]	9.76	0.180	9.95	0.131	8.74	0.055	16.22	0.413	14.07	0.163	11.31	0.105
MSSWD [42]	9.42	0.240	10.62	0.161	9.19	0.059	16.23	0.413	14.10	0.165	11.34	0.106
NLPD [29]	9.23	0.257	9.28	0.162	8.12	0.041	16.21	0.413	14.04	0.162	11.30	0.105
PIEAPP [43]	6.22	<0	7.18	<0	6.61	<0	16.10	0.376	16.03	0.206	12.72	0.096
WADIQAM-FR [44]	9.14	0.231	10.35	0.174	9.06	0.045	16.21	0.414	14.06	0.162	11.30	0.106
TOPIQ-FR [45]	9.31	0.250	9.78	0.168	8.51	0.044	16.22	0.411	14.15	0.165	11.38	0.107

**Table 2:** UVM-NET outputs with 24 images from the O-Haze dataset as the input for each loss, as well as the ground truth (GT).

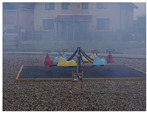	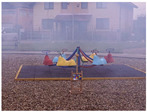	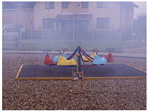	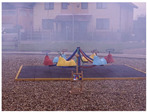
**Hazy Image**	**L2**	**L1**	**Smooth L1**
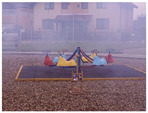	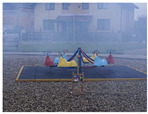	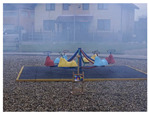	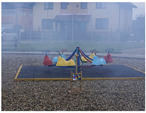
**Huber**	**PSNR**	**SSIM**	**MS-SSIM**
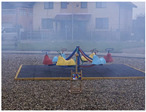	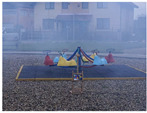	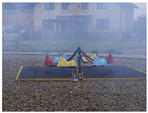	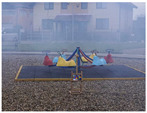
**HaarPSI**	**PSNR-HVS**	**CW-SSIM**	**LPIPS**
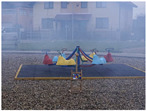	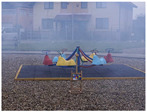	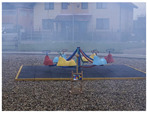	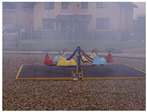
**DISTS**	**MSSWD**	**NLPD**	**PIEAPP**
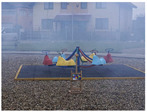	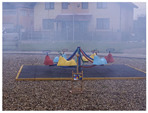	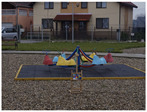	
**WADIQAM-FR**	**TOPIQ-FR**	**GT**	

## Data Availability

No new datasets were created.

## Abbreviations

The following abbreviations are used in this manuscript:

CV	Computer Vision
CNN	Convolutional Neural Network
DCP	Dark Channel Prior
DL	Deep Learning
HVS	Human Visual System
IQA	Image Quality Assessment
PSNR	Peak Signal-to-Noise Ratio
SOTA	State-of-the-art
SSIM	Structural Similarity Index Measure

## References

[1] Hassan H., Mishra P., Ahmad M., Bashir A.K., Huang B., Luo B. (2022). Effects of haze and dehazing on deep learning-based vision models. Appl. Intell..

[2] Panayi S., Artusi A. Hazing or Dehazing: The big dilemma for object detection. Proceedings of the 2021 IEEE 23rd International Workshop on Multimedia Signal Processing (MMSP).

[3] Qiu Y., Lu Y., Wang Y., Jiang H. (2023). IDOD-YOLOV7: Image-Dehazing YOLOV7 for Object Detection in Low-Light Foggy Traffic Environments. Sensors.

[4] He K., Sun J., Tang X. Single image haze removal using dark channel prior. Proceedings of the 2009 IEEE Conference on Computer Vision and Pattern Recognition.

[5] Ancuti C., Ancuti C. (2013). Single image dehazing by multi-scale fusion. IEEE Trans. Image Process..

[6] Ancuti C.O., Ancuti C., Vleeschouwer C.D. Effective local airlight estimation for image dehazing. Proceedings of the 2018 25th IEEE International Conference on Image Processing (ICIP).

[7] Ancuti C.O., Ancuti C., De Vleeschouwer C., Bovick A.C. (2020). Day and Night-Time Dehazing by Local Airlight Estimation. IEEE Trans. Image Process..

[8] Song Y., He Z., Qian H., Du X. (2023). Vision Transformers for Single Image Dehazing. IEEE Trans. Image Process..

[9] Yu H., Huang J., Zheng K., Zhao F. (2024). High-quality Image Dehazing with Diffusion Model. arXiv.

[10] Zheng Z., Wu C. (2024). U-shaped Vision Mamba for Single Image Dehazing. arXiv.

[11] Gu J., Cai H., Dong C., Ren J.S., Qiao Y., Gu S., Timofte R., Cheon M., Yoon S., Kang B. (2021). NTIRE 2021 Challenge on Perceptual Image Quality Assessment, 2021. arXiv.

[12] Gu J., Cai H., Dong C., Ren J.S., Timofte R. (2022). NTIRE 2022 Challenge on Perceptual Image Quality Assessment, 2022. arXiv.

[13] Wandell B. (1995). Foundations of Vision.

[14] Cha S.H. (2007). Comprehensive Survey on Distance/Similarity Measures Between Probability Density Functions. Int. J. Math. Model. Meth. Appl. Sci..

[15] Wang Z., Bovik A.C. (2009). Mean squared error: Love it or leave it? A new look at Signal Fidelity Measures. IEEE Signal Process. Mag..

[16] Wang Z., Simoncelli E., Bovik A. Multiscale structural similarity for image quality assessment. Proceedings of the Thrity-Seventh Asilomar Conference on Signals, Systems & Computers.

[17] Ding K., Ma K., Wang S., Simoncelli E.P. (2021). Comparison of Image Quality Models for Optimization of Image Processing Systems. Int. J. Comput. Vis..

[18] Manheim D., Garrabrant S. (2019). Categorizing Variants of Goodhart’s Law, 2019. arXiv.

[19] Cai B., Xu X., Jia K., Qing C., Tao D. (2016). DehazeNet: An End-to-End System for Single Image Haze Removal. IEEE Trans. Image Process..

[20] Ren W., Pan J., Zhang H., Cao X., Yang M.H. (2020). Single Image Dehazing via Multi-scale Convolutional Neural Networks with Holistic Edges. Int. J. Comput. Vis..

[21] Zhang H., Patel V.M. (2018). Densely Connected Pyramid Dehazing Network. arXiv.

[22] Miao Y., Zhao X., Kan J. (2022). An end-to-end single image dehazing network based on U-net. Signal Image Video Process..

[23] Sutton R. (2019). The Bitter Lesson. Int. J. Math. Model. Methods Appl. Sci..

[24] Zhu H., Peng X., Chandrasekhar V., Li L., Lim J.H. DehazeGAN: When Image Dehazing Meets Differential Programming. Proceedings of the IJCAI.

[25] Fu M., Liu H., Yu Y., Chen J., Wang K. (2021). DW-GAN: A Discrete Wavelet Transform GAN for NonHomogeneous Dehazing. arXiv.

[26] Gui J., Cong X., Cao Y., Ren W., Zhang J., Zhang J., Cao J., Tao D. (2022). A Comprehensive Survey and Taxonomy on Single Image Dehazing Based on Deep Learning 2022. arXiv.

[27] Egiazarian K., Astola J., Lukin V., Battisti F., Carli M. A New Full-Reference Quality Metrics Based on HVS. Proceedings of the Second International Workshop on Video Processing and Quality Metrics.

[28] Wallace G. (1992). The JPEG still picture compression standard. IEEE Trans. Consum. Electron..

[29] Laparra V., Berardino A., Ballé J., Simoncelli E.P. (2017). Perceptually Optimized Image Rendering. J. Opt. Soc. Am. A.

[30] Krizhevsky A., Sutskever I., Hinton G.E. (2012). ImageNet Classification with Deep Convolutional Neural Networks. Proceedings of the Advances in Neural Information Processing Systems.

[31] Simonyan K., Zisserman A. (2015). Very Deep Convolutional Networks for Large-Scale Image Recognition, 2015. arXiv.

[32] Ding K., Ma K., Wang S., Simoncelli E.P. (2020). Image Quality Assessment: Unifying Structure and Texture Similarity. IEEE Trans. Pattern Anal. Mach. Intell..

[33] Zhang R., Isola P., Efros A.A., Shechtman E., Wang O. (2018). The Unreasonable Effectiveness of Deep Features as a Perceptual Metric, 2018. arXiv.

[34] Li B., Peng X., Wang Z., Xu J., Feng D. AOD-Net: All-in-One Dehazing Network. Proceedings of the 2017 IEEE International Conference on Computer Vision (ICCV).

[35] Gu A., Dao T. (2023). Mamba: Linear-Time Sequence Modeling with Selective State Spaces 2023. arXiv.

[36] Wang Q., Ma Y., Zhao K., Tian Y. (2022). A Comprehensive Survey of Loss Functions in Machine Learning. Ann. Data Sci..

[37] Ciampiconi L., Elwood A., Leonardi M., Mohamed A., Rozza A. (2023). A survey and taxonomy of loss functions in machine learning. arXiv.

[38] Wang Z., Bovik A., Sheikh H., Simoncelli E. (2004). Image quality assessment: From error visibility to structural similarity. IEEE Trans. Image Process..

[39] Palubinskas G. Mystery behind similarity measures mse and SSIM. Proceedings of the 2014 IEEE International Conference on Image Processing (ICIP).

[40] Reisenhofer R., Bosse S., Kutyniok G., Wiegand T. (2018). A Haar Wavelet-Based Perceptual Similarity Index for Image Quality Assessment. Signal Process. Image Commun..

[41] Sampat M.P., Wang Z., Gupta S., Bovik A.C., Markey M.K. (2009). Complex Wavelet Structural Similarity: A New Image Similarity Index. IEEE Trans. Image Process..

[42] He J., Wang Z., Wang L., Liu T.I., Fang Y., Sun Q., Ma K. (2024). Multiscale Sliced Wasserstein Distances as Perceptual Color Difference Measures, 2024. arXiv.

[43] Prashnani E., Cai H., Mostofi Y., Sen P. (2018). PieAPP: Perceptual Image-Error Assessment through Pairwise Preference, 2018. arXiv.

[44] Bosse S., Maniry D., Müller K.R., Wiegand T., Samek W. (2018). Deep Neural Networks for No-Reference and Full-Reference Image Quality Assessment. IEEE Trans. Image Process..

[45] Chen C., Mo J., Hou J., Wu H., Liao L., Sun W., Yan Q., Lin W. (2023). TOPIQ: A Top-down Approach from Semantics to Distortions for Image Quality Assessment, 2023. arXiv.

[46] Ponomarenko N., Silvestri F., Egiazarian K., Carli M., Astola J., Lukin V. On between-coefficient contrast masking of DCT basis functions. Proceedings of the 3rd Int Workshop on Video Processing and Quality Metrics for Consumer Electronics.

[47] Ponomarenko N., Ieremeiev O., Lukin V., Egiazarian K., Carli M. Modified image visual quality metrics for contrast change and mean shift accounting. Proceedings of the 2011 11th International Conference The Experience of Designing and Application of CAD Systems in Microelectronics (CADSM).

[48] Rozet F. (2020). PIQA: PyTorch Image Quality Assessement. https://zenodo.org/records/7821605.

[49] Trojanowski K. (2024). lyckantropen/psnr_=_hvsm. Type: Python. https://github.com/lyckantropen/psnr_hvsm.

[50] Chen C. (2025). chaofengc/IQA-PyTorch. original-date: 2021-11-28T13:30:54Z. https://github.com/chaofengc/IQA-PyTorch.

[51] Ancuti C., Ancuti C.O., Timofte R., De Vleeschouwer C. I-HAZE: A dehazing benchmark with real hazy and haze-free indoor images. Proceedings of the International Conference on Advanced Concepts for Intelligent Vision Systems.

[52] Ancuti C.O., Ancuti C., De Vleeschouwer C., Timofte R. O-HAZE: A dehazing benchmark with real hazy and haze-free outdoor images. Proceedings of the IEEE CVPR, NTIRE Workshop.

[53] Ancuti C.O., Ancuti C., Timofte R. NH-HAZE: An Image Dehazing Benchmark with NonHomogeneous Hazy and Haze-Free Images. Proceedings of the IEEE CVPR, NTIRE Workshop.

[54] Ancuti C.O., Ancuti C., Vasluianu F.A., Timofte R. NTIRE 2021 NonHomogeneous Dehazing Challenge Report. Proceedings of the IEEE/CVF Conference on Computer Vision and Pattern Recognition Workshops.

